# Cross-country variation in additive effects of socio-economics, health behaviors, and comorbidities on subjective health of patients with diabetes

**DOI:** 10.1186/2251-6581-13-36

**Published:** 2014-02-21

**Authors:** Shervin Assari

**Affiliations:** 1 Center for Research on Ethnicity, Culture, and Health (CRECH), Department of Health Behavior and Health Education, University of Michigan School of Public Health, 1415 Washington Heights, 2847-SPH I, Ann Arbor, MI 48109-2029, USA

**Keywords:** Subjective health, Socio-economics, Health behaviors, Comorbidity, Cross country study

## Abstract

**Purpose:**

This study explored cross-country differences in the additive effects of socio-economic characteristics, health behaviors and medical comorbidities on subjective health of patients with diabetes.

**Methods:**

The study analyzed data from the Research on Early Life and Aging Trends and Effects (RELATE). The participants were 9,179 adults with diabetes who were sampled from 15 countries (i.e. China, Costa Rica, Puerto Rico, United States, Mexico, Argentina, Barbados, Brazil, Chile, Cuba, Uruguay, India, Ghana, South Africa, and Russia). We fitted three logistic regressions to each country. Model I only included socio-economic characteristics (i.e. age, gender, education and income). In Model II, we also included health behaviors (i.e. smoking, drinking, and exercise). Model III included medical comorbidities (i.e. hypertension, respiratory disease, heart disease, stroke, and arthritis), in addition to the previous blocks.

**Results:**

Our models suggested cross-country differences in the additive effects of socio-economic characteristics, health behaviors and comorbidities on perceived health of patients with diabetes. Comorbid heart disease was the only condition that was consistently associated with poor subjective health regardless of country.

**Conclusion:**

Countries show different profiles of social and behavioral determinants of subjective health among patients with diabetes. Our study suggests that universal programs that assume that determinants of well-being are similar across different countries may be over-simplistic. Thus instead of universal programs that use one protocol for health promotion of patients in all countries, locally designed interventions should be implemented in each country.

## Introduction

It has been consistently shown that individuals with diabetes report poorer well-being and subjective health, compared to people without diabetes [1-5]. A question that is not answered yet is whether poor subjective health of patients with diabetes is the consequence of diabetes - per se - or factors associated with diabetes. We know that low socio-economic status [6], health compromising behaviors [7] and chronic medical conditions [8-12] frequently co-occur with diabetes and also influence the well-being of individuals.

Low socio-economic status may be associated with poor subjective health [6]. The protective effect of high social class on well-being has been partially attributed to better access to financial and material resources available in the community [13]. Unfortunately, most of our knowledge about the effect of socio-economic status on health and well-being of individuals has originated from studies conducted within one country [14,15]. Thus, it is not known if there are cross-country differences in the effect of socio-economic status on subjective health or not.

Comorbid conditions are associated with poor subjective health among patients with an index disease [6]. Patients who suffer from a higher number of chronic conditions tend to report lower physical and mental health related quality of life [16-18]. In the United States, each comorbid chronic condition has been estimated to reduce 3–4 decrements in mental quality of life [19]. Chronic conditions are closely associated with deterioration in physical functioning, physical role, bodily pain, general health, vitality, social functioning, emotional role and mental health [20].

Although research has consistently shown cross-country differences in objective and subjective measures of health [21-26], limited knowledge exists on causes of such variations. The World Values Survey, European Values Study, Eurobarometer, and Latinobarometer, have all reported cross-country variations in self-rated health and wellbeing of individuals [21-33]. It is, however, not known if determinants of well-being also vary based on country. According to our knowledge, there are not many– if any- studies that have compared the effects of social and behavioral determinants of subjective health among individuals with an index chronic medical condition across countries.

The current study aimed to compare countries in the effects of socio-economic characteristics (i.e. age, gender, education and income), health behaviors (i.e. smoking, drinking and exercise), and comorbid conditions (i.e. hypertension, respiratory disease, heart disease, stroke, and arthritis) on the subjective health of a community sample of adults with diabetes.

## Methods

### Study design & participants

Research on Early Life and Aging Trends and Effects (RELATE) is a cross-national survey in 15 countries located in North America, South America, Asia, and Africa [34,35]. The RELATE composed of the following national surveys: 1) China Health and Nutrition Study (CHNS), 2) Chinese Longitudinal Healthy Longevity Survey (CLHLS), 3) Costa Rican Study of Longevity and Healthy Aging (CRELES), 4) Puerto Rican Elderly: Health Conditions (PREHCO), 5) Study of Aging Survey on Health and Well Being of Elders (SABE), 6) WHO Study on Global Ageing and Adult Health (SAGE), and 7) Wisconsin Longitudinal Study (WLS). [34,35] All studies were approved by an institutional review board. Written consent was provided by all participants. Data were collected in an anonymous fashion.

The current analysis included 9,179 adults with diabetes. Participants were sampled in the following 15 countries: China (n = 3,024), Puerto Rico (n = 1,197), the United States (n = 887), Mexico (n = 687), Costa Rica (n = 542), India (n = 478), Brazil (n = 380), South Africa (359), Russia (n = 350), Barbados (n = 325), Cuba (n = 290), Uruguay (n = 188), Chile (n = 173), Ghana (n = 167), and Argentina (n = 132).

The RELATE project represents countries from a diverse range in national income levels. The United States, Puerto Rico, and Barbados represent high income countries; Argentina, Cuba, Uruguay, Chile, Costa Rica, Brazil, Mexico, and Russia represent upper middle income countries; China and India represent lower middle income countries; and Ghana represents low income countries.

### Measures

#### Socio-economic characteristics

The study measured socio-economic data such as age (continuous variable), gender (dichotomous variable), education level (a four level categorical variable composed of no schooling, primary to elementary, secondary to intermediate, and higher), and income (continuous variable).

#### Comorbid conditions

We measured five different chronic medical conditions including hypertension, respiratory disease, heart disease, stroke, and arthritis, using self-report of physician diagnoses. Agreement between self-report and physician diagnosis of comorbid conditions has been shown to be high (kappa: 0.74-0.92) [36].

#### Main outcome

The outcome was a single item measure of subjective health. Overall perceived health was measured using a five-item Likert scale (i.e. very bad, bad, moderate, good, and very good). Single items have been frequently used to measure subjective health and well-being [27,28,37-42]. The test retests reliability of single items for measuring subjective health range from 0.7 to 0.8 [41]. Results of these single item measures of subjective health are highly correlated with standard scales [41,43]. Single item measures of subjective health have shown high predictive validity for prediction of mortality, even after controlling for other risk factors [29].

### Data analysis

Data analysis was conducted using SPSS 20.0 for Windows. We transformed our five-item Likert scale to a dichotomous outcome, as poor health (i.e. very bad health and bad health) versus good health (i.e. moderate health, good health, and very good health). Odds Ratios (OR) and 95% confidence intervals (95% CI) were reported. *P* less than 0.05 was considered as significant.

We fitted country specific logistic regressions to determine if the associations between socio-economic factors (i.e age, gender, education, and income), health behaviors (i.e. smoking, drinking, and exercise) and chronic conditions (i.e. hypertension, respiratory disease, heart disease, stroke, and arthritis), and subjective health vary across countries. Although most country specific surveys had sampling weights, sampling weights were not applicable to surveys from the United States (Wisconsin) and China (CHNS). Thus, the current study did not apply sampling weights.

We took a hierarchical approach for our regression analysis. Model I only included socio-economic characteristics (i.e. age, gender, education and income). In Model II, health behaviors (i.e. smoking, drinking, and exercise) were added to the model. Model III also included comorbidities (i.e. hypertension, respiratory disease, heart disease, stroke, and arthritis).

Changes in the odds ratios from Model I (socio-economic factors) to Model II (socio-economic factors and health behaviors) suggest that health behaviors may mediate the effect of socio-economic factors on subjective health. Changes in the odds ratios from Model II (socio-economic factors and health behaviors) to Model III (full model) suggest that comorbid conditions may mediate the effect of socio-economic factors and health behaviors on subjective health.

## Results

This study included 9,179 adults with diabetes. Participants were sampled in the following 15 countries: China (n = 3,024), Puerto Rico (n = 1,197), the United States (n = 887), Mexico (n = 687), Costa Rica (n = 542), India (n = 478), Brazil (n = 380), South Africa (359), Russia (n = 350), Barbados (n = 325), Cuba (n = 290), Uruguay (n = 188), Chile (n = 173), Ghana (n = 167), and Argentina (n = 132).

### Model I (socio-economics)

With the exception of Costa Rica, the United States, Mexico, Brazil, and South Africa, in all 10 other countries, female patients had significantly poorer subjective health than male patients [Table 1].

**Table 1:** **Socio**-**economic predictors of poor subjective health among patients with diabetes in 15 countries**

	**B**	**S.E.**	**Wald**	**Sig.**	**Exp (B)**	**95% C.I. for EXP (B)**	
						**Lower**	**Upper**
**China**							
Female	.183	.028	41.441	<.001	1.201	1.136	1.269
Age	−.016	.001	334.036	<.001	.984	.982	.986
Education	−.211	.016	176.776	<.001	.810	.785	.835
Income	.000	.000	178.850	<.001	1.000	1.000	1.000
**Costa Rica**							
Female	.121	.083	2.116	.146	1.129	.959	1.328
Age	−.014	.004	12.238	<.001	.986	.978	.994
Education	−.378	.068	31.278	<.001	.685	.600	.782
Income	.000	.000	10.246	.001	1.000	1.000	1.000
**Puerto Rico**							
Female	.487	.075	42.085	<.001	1.628	1.405	1.886
Age	−.004	.005	.630	.427	.996	.987	1.005
Education	−.462	.050	85.795	<.001	.630	.572	.695
Income	.000	.000	17.886	<.001	1.000	1.000	1.000
**United States**							
Female	−.105	.082	1.636	.201	.901	.767	1.057
Age	.060	.055	1.198	.274	1.062	.953	1.183
Education	−.517	.102	25.588	<.001	.596	.488	.728
Income	.000	.000	23.914	<.001	1.000	1.000	1.000
**Mexico**							
Female	.105	.080	1.691	.193	1.110	.948	1.300
Age	.016	.005	12.286	<.001	1.016	1.007	1.025
Education	−.305	.054	32.476	<.001	.737	.664	.819
Income	.000	.000	17.668	<.001	1.000	1.000	1.000
**Argentina**							
Female	.363	.155	5.494	.019	1.438	1.061	1.949
Age	−.013	.010	1.718	.190	.987	.967	1.007
Education	−.763	.104	53.394	<.001	.466	.380	.572
Income	.000	.000	2.467	.116	1.000	1.000	1.000
**Barbados**							
Female	.407	.120	11.421	.001	1.502	1.186	1.901
Age	.041	.007	31.863	<.001	1.042	1.027	1.057
Education	−.290	.099	8.624	.003	.748	.617	.908
Income	.000	.000	4.121	.042	1.000	1.000	1.000
**Brazil**							
Female	.040	.090	.192	.661	1.040	.872	1.241
Age	.001	.005	.045	.832	1.001	.991	1.012
Education	−.279	.063	19.373	<.001	.756	.668	.856
Income	.000	.000	17.582	<.001	1.000	1.000	1.000
**Chile**							
Female	.351	.125	7.875	.005	1.421	1.112	1.816
Age	.003	.008	.153	.696	1.003	.988	1.018
Education	−.326	.063	26.812	<.001	.722	.638	.817
Income	.000	.000	.016	.899	1.000	1.000	1.000
**Cuba**							
Female	.531	.103	26.484	<.001	1.701	1.389	2.082
Age	−.005	.006	.623	.430	.995	.983	1.007
Education	−.317	.075	18.155	<.001	.728	.629	.842
Income	.000	.000	1.871	.171	1.000	1.000	1.000
**Uruguay**							
Female	.387	.124	9.774	.002	1.472	1.155	1.876
Age	−.001	.008	.005	.945	.999	.984	1.015
Education	−.404	.070	32.948	<.001	.667	.581	.766
Income	.000	.000	1.744	.187	1.000	1.000	1.000
**India**							
Female	.176	.069	6.487	.011	1.192	1.041	1.364
Age	.047	.003	193.134	<.001	1.048	1.041	1.055
Education	−.213	.041	26.517	<.001	.808	.746	.877
Income	.000	.000	17.654	<.001	1.000	1.000	1.000
**Ghana**							
Female	.263	.105	6.257	.012	1.301	1.059	1.598
Age	.055	.005	135.610	<.001	1.056	1.047	1.066
Education	−.129	.055	5.598	.018	.879	.789	.978
Income	.000	.000	.132	.716	1.000	1.000	1.000
**South Africa**							
Female	.057	.102	.306	.580	1.058	.866	1.293
Age	.025	.005	24.866	<.001	1.025	1.015	1.035
Education	−.061	.034	3.120	.077	.941	.880	1.007
Income	.000	.000	2.535	.111	1.000	1.000	1.000
**Russia**							
Female	.277	.099	7.854	.005	1.319	1.087	1.602
Age	.074	.005	214.090	<.001	1.077	1.067	1.088
Education	−.261	.073	12.717	<.001	.771	.668	.889
Income	.000	.000	16.061	<.001	1.000	1.000	1.000

In six countries (i.e. Mexico, Barbados, India, Ghana, South Africa, and Russia), older patients had poorer subjective health than younger patients. In China and Costa Rica, older patients reported better subjective health. In the other seven countries (i.e. Puerto Rico, the United States, Brazil, Chile, Cuba, Argentina, and Uruguay), age was not associated with subjective health [Table 1].

In all countries other than South Africa, high education was associated with better subjective health. This association was marginally significant in South Africa [Table 1].

In six countries (i.e. Argentina, Chile, Cuba, Uruguay, Ghana, and South Africa), high income was not associated with subjective health. High income was predictive of better subjective health in the other nine countries [Table 1].

### Model II (socio-economics and health behaviors)

In all countries but Mexico, exercise was predictive of better subjective health. In Mexico, exercise was associated with worse subjective health [Table 2].

**Table 2:** Socio-economics, behaviors, and number of chronic conditions as predictors of poor subjective health among patients with diabetes in 15 countries

	**B**	**S.E.**	**Wald**	**Sig.**	**Exp (B)**	**95% C.I. for EXP (B)**	
						**Lower**	**Upper**
**China**							
Female	.139	.037	13.854	<.001	1.149	1.068	1.236
Age	−.016	.001	284.715	<.001	.985	.983	.986
Education	−.203	.017	139.722	<.001	.817	.790	.844
Income	.000	.000	192.184	<.001	1.000	1.000	1.000
Smoking	.106	.038	7.674	.006	1.112	1.031	1.198
Drinking	−.153	.035	18.984	<.001	.858	.802	.919
Exercising	−.377	.031	146.203	<.001	.686	.645	.729
**Costa Rica**							
Female	.029	.109	.071	.790	1.030	.831	1.276
Age	−.019	.004	20.334	<.001	.981	.973	.989
Education	−.394	.069	32.336	<.001	.674	.588	.772
Income	.000	.000	8.779	.003	1.000	1.000	1.000
Smoking	.011	.099	.011	.915	1.011	.833	1.226
Drinking	−.010	.109	.009	.924	.990	.799	1.226
Exercising	−.590	.105	31.737	<.001	.554	.452	.681
**Puerto Rico**							
Female	.461	.084	29.913	.000	1.585	1.344	1.870
Age	−.011	.005	5.302	.021	.989	.980	.998
Education	−.401	.051	62.523	<.001	.669	.606	.739
Income	.000	.000	14.095	.000	1.000	1.000	1.000
Smoking	.283	.086	10.753	.001	1.327	1.120	1.571
Drinking	−.336	.102	10.931	.001	.714	.585	.872
Exercising	−.448	.078	32.801	<.001	.639	.548	.745
**United States**							
Female	−.054	.097	.306	.580	.948	.784	1.146
Age	.049	.066	.557	.455	1.051	.923	1.196
Education	−.333	.116	8.273	.004	.717	.571	.899
Income	.000	.000	12.963	<.001	1.000	1.000	1.000
Smoking	.604	.102	35.374	<.001	1.830	1.500	2.233
Drinking	−.703	.097	52.461	<.001	.495	.409	.599
Exercising	−1.056	.200	28.031	<.001	.348	.235	.514
**Mexico**							
Female	.023	.100	.055	.815	1.024	.841	1.246
Age	.017	.005	11.807	.001	1.017	1.007	1.026
Education	−.291	.055	27.461	<.001	.748	.671	.834
Income	.000	.000	16.775	<.001	1.000	1.000	1.000
Smoking	.462	.096	23.380	<.001	1.588	1.316	1.915
Drinking	−1.108	.099	125.824	<.001	.330	.272	.401
Exercising	.546	.102	28.673	<.001	1.727	1.414	2.109
**Argentina**							
Female	.374	.182	4.222	.040	1.453	1.017	2.075
Age	−.014	.011	1.657	.198	.986	.966	1.007
Education	−.756	.108	49.389	<.001	.470	.380	.580
Income	.000	.000	2.127	.145	1.000	1.000	1.000
Smoking	.415	.172	5.853	.016	1.515	1.082	2.120
Drinking	−.528	.160	10.903	.001	.590	.431	.807
Exercising	−.622	.243	6.541	.011	.537	.333	.865
**Barbados**							
Female	.330	.147	5.028	.025	1.390	1.042	1.855
Age	.032	.008	17.359	<.001	1.032	1.017	1.048
Education	−.273	.103	7.082	.008	.761	.622	.931
Income	.000	.000	3.758	.053	1.000	1.000	1.000
Smoking	.154	.160	.921	.337	1.166	.852	1.597
Drinking	−.564	.143	15.517	<.001	.569	.429	.753
Exercising	−.503	.124	16.409	<.001	.605	.474	.771
**Brazil**							
Female	.012	.108	.012	.913	1.012	.819	1.250
Age	−.007	.006	1.470	.225	.993	.982	1.004
Education	−.196	.065	9.063	.003	.822	.723	.934
Income	.000	.000	11.466	.001	1.000	1.000	1.000
Smoking	.397	.104	14.675	<.001	1.488	1.214	1.823
Drinking	−.788	.105	56.162	<.001	.455	.370	.559
Exercising	−.680	.111	37.302	<.001	.507	.407	.630
**Chile**							
Female	.253	.136	3.475	.062	1.288	.987	1.682
Age	.001	.008	.021	.885	1.001	.986	1.016
Education	−.323	.064	25.809	<.001	.724	.639	.820
Income	.000	.000	.000	.989	1.000	1.000	1.000
Smoking	.179	.128	1.943	.163	1.196	.930	1.537
Drinking	−.395	.130	9.271	.002	.674	.523	.869
Exercising	−.408	.146	7.809	.005	.665	.499	.885
**Cuba**							
Female	.472	.119	15.580	<.001	1.603	1.268	2.025
Age	−.008	.006	1.389	.239	.992	.980	1.005
Education	−.264	.076	12.158	<.001	.768	.662	.891
Income	.000	.000	1.217	.270	1.000	1.000	1.000
Smoking	.251	.115	4.785	.029	1.285	1.026	1.609
Drinking	−.434	.127	11.570	.001	.648	.505	.832
Exercising	−.382	.119	10.371	.001	.682	.541	.861
**Uruguay**							
Female	.201	.149	1.805	.179	1.222	.912	1.639
Age	−.006	.008	.581	.446	.994	.978	1.010
Education	−.366	.072	25.639	<.001	.693	.602	.799
Income	.000	.000	.887	.346	1.000	1.000	1.000
Smoking	.180	.140	1.668	.197	1.198	.911	1.575
Drinking	−.682	.132	26.538	<.001	.506	.390	.656
Exercising	−.809	.194	17.446	<.001	.445	.305	.651
**India**							
Female	.293	.080	13.231	<.001	1.340	1.145	1.569
Age	.040	.004	129.415	<.001	1.041	1.034	1.048
Education	−.205	.042	23.824	<.001	.814	.750	.884
Income	.000	.000	15.854	<.001	1.000	1.000	1.000
Smoking	.337	.072	21.774	<.001	1.401	1.216	1.614
Drinking	.166	.095	3.037	.081	1.181	.980	1.423
Exercising	−.613	.077	63.331	<.001	.542	.466	.630
**Ghana**							
Female	.284	.119	5.655	.017	1.328	1.051	1.679
Age	.052	.005	115.199	<.001	1.053	1.043	1.063
Education	−.188	.056	11.171	.001	.829	.742	.925
Income	.000	.000	.160	.689	1.000	1.000	1.000
Smoking	.236	.135	3.037	.081	1.266	.971	1.651
Drinking	.165	.109	2.307	.129	1.180	.953	1.460
Exercising	−.587	.108	29.316	<.001	.556	.449	.687
**South Africa**							
Female	.064	.108	.348	.555	1.066	.863	1.316
Age	.025	.005	22.845	<.001	1.025	1.015	1.035
Education	−.052	.036	2.075	.150	.950	.885	1.019
Income	.000	.000	2.049	.152	1.000	1.000	1.000
Smoking	.156	.122	1.643	.200	1.169	.921	1.484
Drinking	.219	.131	2.816	.093	1.245	.964	1.608
Exercising	−.665	.179	13.800	<.001	.515	.362	.731
**Russia**							
Female	.372	.131	8.002	.005	1.450	1.121	1.876
Age	.070	.005	175.456	<.001	1.073	1.062	1.084
Education	−.256	.075	11.785	.001	.774	.669	.896
Income	.000	.000	14.406	<.001	1.000	1.000	1.000
Smoking	.417	.140	8.907	.003	1.518	1.154	1.996
Drinking	−.146	.111	1.725	.189	.864	.695	1.074
Exercising	−.746	.118	40.223	<.001	.474	.377	.597

In India and South Africa, drinking was marginally associated with poor subjective health. In Ghana, and Russia, drinking was not associated with subjective health. In all other 12 countries, drinking was associated with better subjective health [Table 2].

In Ghana, smoking was marginally associated with poor subjective health. In Costa Rica, Barbados, Chile, Uruguay, and South Africa, smoking was not associated with subjective health. In all other nine countries, smoking was associated with poor subjective health [Table 2].

### Model III (socio-economics, health behaviors and comorbidities)

With no exception, comorbid heart disease was associated with poor subjective health in all countries. With an exception of South Africa, in all other countries, comorbid hypertension was associated with poor subjective health. Arthritis was associated with poor subjective health in all countries but Ghana. In countries other than China and Ghana, comorbid lung disease was associated with poor subjective health. With an exception of China, Argentina and Ghana, in all other countries, stroke was associated with poor subjective health. In Ghana, the association between stroke and subjective health was marginally significant [Table 3].

**Table 3:** Socio-economics, behaviors and chronic conditions as predictors of poor subjective health among patients with diabetes in 15 countries

	**B**	**S.E.**	**Wald**	**Sig.**	**Exp (B)**	**95% C.I. for EXP (B)**	
						**Lower**	**Upper**
**China**							
Female	.145	.046	9.782	.002	1.156	1.056	1.267
Age	−.003	.001	6.835	.009	.997	.994	.999
Education	−.185	.026	50.921	<.001	.831	.790	.875
Income	.000	.000	87.633	<.001	1.000	1.000	1.000
Smoking	.217	.047	21.218	<.001	1.242	1.133	1.362
Drinking	−.156	.043	12.915	<.001	.856	.786	.932
Exercising	−.563	.040	196.258	<.001	.570	.527	.616
Hypertension	.232	.045	26.124	<.001	1.261	1.154	1.378
Lung Disease	.048	.057	.712	.399	1.049	.939	1.172
Heart Disease	.527	.055	91.014	<.001	1.694	1.520	1.888
Stroke	−.054	.070	.585	.445	.948	.826	1.087
Arthritis	.431	.046	86.767	<.001	1.539	1.406	1.685
**Costa Rica**							
Female	−.074	.114	.421	.517	.929	.743	1.161
Age	−.021	.004	21.239	<.001	.980	.971	.988
Education	−.448	.072	38.379	<.001	.639	.554	.736
Income	.000	.000	6.267	.012	1.000	1.000	1.000
Smoking	−.027	.102	.070	.791	.973	.797	1.189
Drinking	.016	.113	.020	.888	1.016	.814	1.267
Exercising	−.497	.108	21.128	<.001	.608	.492	.752
Hypertension	.272	.088	9.463	.002	1.312	1.104	1.560
Lung Disease	.485	.117	17.282	<.001	1.624	1.292	2.041
Heart Disease	.501	.131	14.612	<.001	1.650	1.276	2.133
Stroke	.375	.191	3.871	.049	1.456	1.001	2.116
Arthritis	.433	.119	13.354	<.001	1.542	1.222	1.946
**Puerto Rico**							
Female	.277	.090	9.399	.002	1.319	1.105	1.575
Age	−.019	.005	13.947	<.001	.981	.971	.991
Education	−.407	.053	58.032	<.001	.666	.599	.739
Income	.000	.000	15.183	<.001	1.000	1.000	1.000
Smoking	.242	.091	7.089	.008	1.274	1.066	1.523
Drinking	−.184	.107	2.959	.085	.832	.674	1.026
Exercising	−.353	.083	18.283	<.001	.702	.597	.826
Hypertension	.664	.080	68.161	<.001	1.943	1.660	2.275
Lung Disease	.576	.183	9.964	.002	1.779	1.244	2.545
Heart Disease	.826	.123	45.129	<.001	2.285	1.796	2.908
Stroke	.590	.212	7.753	.005	1.805	1.191	2.734
Arthritis	.818	.083	97.363	<.001	2.265	1.926	2.665
**United States**							
Female	.020	.108	.033	.855	1.020	.825	1.260
Age	.033	.071	.220	.639	1.034	.900	1.187
Education	−.273	.124	4.809	.028	.761	.596	.971
Income	.000	.000	12.312	<.001	1.000	1.000	1.000
Smoking	.417	.110	14.458	<.001	1.517	1.224	1.881
Drinking	−.527	.106	24.865	<.001	.590	.480	.726
Exercising	−1.086	.212	26.201	<.001	.337	.223	.511
Hypertension	.489	.104	21.986	<.001	1.630	1.329	1.999
Lung Disease	.759	.118	41.048	<.001	2.135	1.693	2.693
Heart Disease	1.361	.109	157.177	<.001	3.902	3.154	4.827
Stroke	1.035	.195	28.045	<.001	2.816	1.920	4.131
Arthritis	.685	.104	43.091	<.001	1.984	1.617	2.435
**Mexico**							
Female	−.201	.107	3.517	.061	.818	.663	1.009
Age	.013	.005	6.592	.010	1.013	1.003	1.024
Education	−.310	.058	28.134	<.001	.734	.654	.823
Income	.000	.000	15.857	<.001	1.000	1.000	1.000
Smoking	.385	.101	14.426	<.001	1.469	1.205	1.792
Drinking	−1.192	.105	129.940	<.001	.303	.247	.373
Exercising	.587	.106	30.569	<.001	1.799	1.461	2.215
Hypertension	.349	.089	15.502	<.001	1.418	1.192	1.687
Lung Disease	.734	.161	20.753	<.001	2.083	1.519	2.857
Heart Disease	.285	.137	4.331	.037	1.329	1.017	1.738
Stroke	.443	.189	5.485	.019	1.557	1.075	2.256
Arthritis	1.018	.111	84.795	<.001	2.768	2.229	3.438
**Argentina**							
Female	.172	.201	.732	.392	1.188	.801	1.760
Age	−.024	.012	4.206	.040	.976	.954	.999
Education	−.736	.116	40.039	<.001	.479	.381	.602
Income	.000	.000	2.969	.085	1.000	1.000	1.000
Smoking	.446	.187	5.701	.017	1.562	1.083	2.251
Drinking	−.519	.173	9.005	.003	.595	.424	.835
Exercising	−.394	.259	2.302	.129	.675	.406	1.122
Hypertension	.548	.161	11.643	.001	1.729	1.263	2.369
Lung Disease	1.283	.289	19.658	<.001	3.607	2.046	6.358
Heart Disease	.956	.194	24.405	<.001	2.603	1.781	3.804
Stroke	.428	.383	1.248	.264	1.534	.724	3.248
Arthritis	.999	.169	34.967	<.001	2.716	1.950	3.782
**Barbados**							
Female	.021	.161	.016	.898	1.021	.744	1.400
Age	.032	.008	15.068	<.001	1.032	1.016	1.049
Education	−.283	.108	6.806	.009	.754	.610	.932
Income	.000	.000	4.073	.044	1.000	1.000	1.000
Smoking	.023	.171	.018	.894	1.023	.732	1.430
Drinking	−.503	.152	10.996	.001	.605	.449	.814
Exercising	−.372	.132	7.919	.005	.690	.532	.893
Hypertension	.565	.129	19.170	<.001	1.759	1.366	2.264
Lung Disease	1.248	.349	12.774	<.001	3.482	1.757	6.903
Heart Disease	.641	.208	9.530	.002	1.898	1.263	2.850
Stroke	.918	.313	8.587	.003	2.504	1.355	4.628
Arthritis	.810	.129	39.233	<.001	2.247	1.744	2.895
**Brazil**							
Female	−.101	.116	.750	.386	.904	.720	1.135
Age	−.009	.006	2.442	.118	.991	.979	1.002
Education	−.218	.068	10.133	.001	.804	.704	.920
Income	.000	.000	9.953	.002	1.000	1.000	1.000
Smoking	.392	.109	12.853	<.001	1.481	1.195	1.835
Drinking	−.709	.111	40.701	<.001	.492	.396	.612
Exercising	−.555	.117	22.487	<.001	.574	.457	.722
Hypertension	.560	.097	33.052	<.001	1.751	1.447	2.120
Lung Disease	.494	.151	10.666	.001	1.638	1.218	2.203
Heart Disease	.622	.127	24.113	<.001	1.862	1.453	2.386
Stroke	.514	.197	6.777	.009	1.672	1.135	2.461
Arthritis	.676	.106	40.333	<.001	1.965	1.595	2.421
**Chile**							
Female	.080	.148	.290	.590	1.083	.811	1.447
Age	−.012	.008	2.218	.136	.988	.972	1.004
Education	−.332	.066	25.001	<.001	.717	.630	.817
Income	.000	.000	.110	.740	1.000	1.000	1.000
Smoking	.100	.135	.547	.460	1.105	.848	1.441
Drinking	−.328	.137	5.737	.017	.721	.551	.942
Exercising	−.417	.155	7.280	.007	.659	.487	.892
Hypertension	.699	.129	29.203	<.001	2.012	1.561	2.592
Lung Disease	.911	.227	16.179	<.001	2.488	1.596	3.879
Heart Disease	.360	.139	6.658	.010	1.433	1.090	1.883
Stroke	.656	.298	4.838	.028	1.928	1.074	3.460
Arthritis	.627	.148	17.868	<.001	1.873	1.400	2.505
**Cuba**							
Female	.080	.133	.362	.548	1.083	.835	1.404
Age	−.006	.007	.826	.363	.994	.980	1.008
Education	−.292	.082	12.576	<.001	.747	.636	.878
Income	.000	.000	.665	.415	1.000	1.000	1.000
Smoking	.207	.124	2.785	.095	1.230	.964	1.570
Drinking	−.386	.139	7.770	.005	.680	.518	.892
Exercising	−.483	.129	13.956	<.001	.617	.479	.795
Hypertension	.550	.118	21.728	<.001	1.733	1.375	2.183
Lung Disease	.794	.192	17.158	<.001	2.211	1.519	3.219
Heart Disease	1.150	.158	53.301	<.001	3.158	2.319	4.300
Stroke	.512	.226	5.134	.023	1.669	1.072	2.598
Arthritis	1.068	.114	87.228	.000	2.909	2.325	3.639
**Uruguay**							
Female	.092	.164	.314	.575	1.096	.795	1.512
Age	−.011	.009	1.567	.211	.989	.971	1.006
Education	−.396	.078	26.041	<.001	.673	.578	.784
Income	.000	.000	.278	.598	1.000	1.000	1.000
Smoking	.166	.150	1.230	.267	1.181	.880	1.584
Drinking	−.592	.142	17.408	<.001	.553	.419	.731
Exercising	−.660	.206	10.220	.001	.517	.345	.775
Hypertension	.491	.131	13.954	<.001	1.634	1.263	2.113
Lung Disease	1.212	.221	30.110	<.001	3.362	2.180	5.183
Heart Disease	.807	.151	28.710	<.001	2.241	1.668	3.010
Stroke	1.012	.332	9.282	.002	2.752	1.435	5.278
Arthritis	.749	.132	32.109	<.001	2.114	1.632	2.739
**India**							
Female	.147	.093	2.491	.115	1.158	.965	1.390
Age	.035	.004	71.757	<.001	1.035	1.027	1.044
Education	−.271	.049	31.218	<.001	.762	.693	.839
Income	.000	.000	9.795	.002	1.000	1.000	1.000
Smoking	.349	.083	17.567	<.001	1.418	1.204	1.669
Drinking	.029	.112	.068	.794	1.030	.826	1.283
Exercising	−.695	.091	58.313	<.001	.499	.417	.596
Hypertension	.460	.093	24.401	<.001	1.585	1.320	1.902
Lung Disease	.785	.156	25.286	<.001	2.193	1.615	2.978
Heart Disease	.705	.083	71.269	<.001	2.023	1.718	2.383
Stroke	.670	.210	10.211	.001	1.954	1.296	2.946
Arthritis	.555	.087	40.684	<.001	1.742	1.469	2.065
**Ghana**							
Female	.331	.128	6.678	.010	1.392	1.083	1.789
Age	.055	.005	116.455	<.001	1.057	1.046	1.068
Education	−.182	.061	9.080	.003	.833	.740	.938
Income	.000	.000	.131	.717	1.000	1.000	1.000
Smoking	.288	.144	3.991	.046	1.333	1.005	1.768
Drinking	.177	.115	2.371	.124	1.193	.953	1.494
Exercising	−.530	.115	21.227	<.001	.588	.470	.737
Hypertension	.373	.144	6.766	.009	1.453	1.096	1.925
Lung Disease	−.097	.659	.021	.883	.908	.250	3.301
Heart Disease	.391	.150	6.814	.009	1.479	1.102	1.985
Stroke	.526	.270	3.792	.052	1.691	.997	2.871
Arthritis	−.208	.145	2.057	.152	.812	.611	1.079
**South Africa**							
Female	.045	.116	.151	.698	1.046	.833	1.314
Age	.023	.006	16.941	<.001	1.023	1.012	1.034
Education	−.054	.038	2.024	.155	.947	.879	1.021
Income	.000	.000	1.156	.282	1.000	1.000	1.000
Smoking	.068	.130	.273	.601	1.070	.829	1.381
Drinking	.299	.140	4.564	.033	1.349	1.025	1.776
Exercising	−.663	.192	11.969	.001	.515	.354	.750
Hypertension	.028	.118	.057	.812	1.029	.816	1.297
Lung Disease	1.205	.267	20.325	<.001	3.335	1.976	5.631
Heart Disease	.706	.162	18.995	<.001	2.026	1.475	2.783
Stroke	1.279	.236	29.486	<.001	3.594	2.265	5.702
Arthritis	.738	.120	37.630	<.001	2.092	1.653	2.649
**Russia**							
Female	.331	.159	4.314	.038	1.392	1.019	1.903
Age	.050	.006	60.756	<.001	1.051	1.038	1.064
Education	−.277	.088	10.041	.002	.758	.638	.900
Income	.000	.000	16.812	<.001	1.000	1.000	1.000
Smoking	.509	.169	9.072	.003	1.664	1.195	2.318
Drinking	−.306	.132	5.355	.021	.737	.569	.954
Exercising	−.670	.138	23.611	<.001	.512	.390	.670
Hypertension	.296	.128	5.345	.021	1.344	1.046	1.727
Lung Disease	.376	.137	7.508	.006	1.456	1.113	1.905
Heart Disease	1.140	.119	91.247	<.001	3.126	2.474	3.949
Stroke	.846	.206	16.849	<.001	2.330	1.556	3.490
Arthritis	.690	.114	36.867	<.001	1.993	1.595	2.490

## Discussion

The purpose of this study was to explore cross-country differences in the associations between socio-economic characteristics, health behaviors and comorbid medical conditions with subjective health among individuals with diabetes. The study showed that low socio-economic status, smoking, lack of exercise, and medical comorbidities are predictive of poor subjective health of patients with diabetes in most countries. The study, however, documented several cross–country differences in the links between socio-economics, health behaviors and chronic conditions, and subjective health of individuals with diabetes. The only factor with a consistent effect on subjective health of patients with diabetes was comorbid heart disease. These findings suggest that the link between social and behavioral determinants of health and subjective health may vary across countries.

With exception of the United States, Costa Rica, Mexico, Brazil, and South Africa, in all ten other countries, female gender was associated with poor subjective health among individuals with diabetes. According to another study among the general population, in 6 of 15 countries (i.e. China, Costa Rica, Puerto Rico, Barbados, Cuba and Uruguay) women reported poorer subjective health than men [44]. Among individuals with at least one chronic medical condition in Uruguay, Ghana and South Africa, female gender was associated with worse subjective health. Gender was not associated with subjective health in other countries [45]. These findings explain the complex role of gender in shaping the well-being of individuals. These studies collectively suggest that there are variations in the effect of gender on well-being between various populations, and sometimes even within a single country. The effect of gender on health and well-being among patients with medical conditions may be different from gender's effects among the general population. Interestingly, the role of gender on the well-being of patients with medical conditions may depend on type of chronic illness.

Literature suggests that women tend to report a higher number of self-reported chronic medical conditions and poorer self-reported health [46]. Women also report worse subjective health and well-being, compared to men [46]. Due to gender differences in longevity, a larger part of a woman’s life is spent with illness and disabilities [47]. Although women require more care later in life than men, women tend to have less access to health resources [48,49]. In Ghana and Uruguay, among individuals with one chronic medical condition, women were more vulnerable to the effect of education on subjective health [45]. In a study on patients with chronic heart disease from Iran, women were more prone to the effect of income and education on sleep quality [50].

Pinquart and Sörensen proposed a number of mechanisms that may explain gender differences in subjective well-being. First, due to gender inequities and gendered social power, women may have lower material resources. In several countries, the gendered labor market may result in a lower level of stable employment among women [51]. Even among those who are employed, women’s pensions may be lower than men’s [52]. Among elderly, women more frequently live in poverty compared to men [53]. In addition, older women are more likely to be widowed than men [53]. In the United States, nearly four times as many older women than men live alone [49]. Finally, gender differences in response sets may explain worse self-reported health among women, as women may have more tendencies to report negative feelings and emotions [54].

Our results suggested that age and subjective well-being of patients with diabetes may be differently linked across countries. While in a number of countries (i.e. Mexico, Barbados, India, Ghana, South Africa, and Russia) high age is predictive of poor subjective health, age may not be associated with subjective health of patients with diabetes in other countries (i.e. Puerto Rico, United States, Brazil, Chile, Cuba, Argentina, and Uruguay). Interestingly, in China and Costa Rica, high age was associated with better subjective health among patients with diabetes. A recent study of general populations showed that in three countries (i.e. China, Costa Rica and Argentina), high age may predict better subjective health, while in four countries (i.e. Barbados , India, South Africa and Russia), high age was associated with low subjective health. Based on that study, in seven countries (i.e. Puerto Rico, United States, Mexico, Brazil, Chile, Cuba and Uruguay), a linear association between age and subjective health of elderly individuals in the general population could not be found [44]. Among individuals with at least one chronic medical condition, high age was associated with better subjective health in China, Costa Rica, Puerto Rico, Brazil and Argentina. In that study, high age was associated with poor subjective health in India, Ghana, South Africa and Russia. Age and subjective health were not significantly associated in other countries [45]. There are studies suggesting that there is an improvement in well-being as age increases among older individuals [55,56]. A study among patients with heart disease showed that patients older than 65 years had better health-related quality of life than those younger [45].

Based on Model I, low education was consistently associated with higher risk of poor subjective health among patients with diabetes. Based on a recent study among general populations, education was not associated with subjective health in the United States, Ghana or South Africa [44]. Among patients with chronic conditions, education was not associated with subjective health in the United States, Mexico, Barbados, Brazil, Uruguay, Ghana, South Africa, or Russia. [45] The effect of education on health and well-being might be due to income or marital status [57]. Other reasons that highly educated people may stay healthier include social support and health protective behaviors [57].

Based on our study, in nine countries, income had an effect on subjective health of patients with diabetes, above and beyond the effect of education and other socio-economic factors. In Argentina, Chile, Cuba, Uruguay, Ghana, and South Africa, income did not have an effect on subjective health of patients with diabetes while the effect of education was controlled. Similar results were reported on the residual effect of income after controlling education in nine of 15 countries by a study that included a general population [44]. Among patients with at least one chronic medical condition, income was not predictive of poor subjective health in Argentina, Chile, Cuba, India, Ghana, or South Africa [45]. In India, the effect of income on subjective health of patients with chronic medical conditions was larger among women than men [45]. In Iran, among patients with chronic heart disease, the effect of income on well-being was larger for women than men [50]. These findings suggest that the links between country, gender, education, income and well-being are very complex.

A recent study suggested that the complex interplay between socio-economic status, chronic conditions and subjective health varies from setting to setting. In the United States, chronic conditions may explain the effect of marital status on health, while in Puerto Rico, the effect of income on subjective health was attributed to chronic conditions. In Costa Rica, Argentina, Barbados, Cuba, and Uruguay, chronic conditions explained gender disparities in subjective health. In China, Mexico, Brazil, Russia, Chile, India, Ghana and South Africa, the effect of socio-economic status was not due to chronic conditions [44].

Based on our study, comorbid heart disease was consistently predictive of poor subjective health among patients with diabetes. The effects of other chronic conditions on subjective health, however, were moderated by country. A study among 21,133 individuals on the association between number of chronic somatic conditions and quality of life showed an association between presence of a chronic condition and lower well-being across all domains of subjective health including physical function, fatigue, pain, emotional distress, and social function. Presence of two or more conditions was associated with larger decrements in quality of life, compared to a single condition [58]. Another large study among adults showed that after adjustments for socio-economic status and health behaviors (i.e. smoking, alcohol consumption, and physical activity), people with 3 or more chronic medical conditions were more likely to report poor general health, mental distress, physical distress, and activity limitations compared to individuals who had one or two chronic conditions [59,60].

Our study may have important implications for global public health policy and practice. As countries show different sets of determinants of subjective health among individuals, we suggest that country should be considered as the context that shapes social and behavioral determinants of health. Comorbid heart disease, however, has a consistent effect and should be universally diagnosed and treated among patients with diabetes. Thus, we do not recommend universal programs for health promotion of patients with diabetes across countries. Based on our findings, tailored health promotion programs should be designed specific to each country.

Universal programs focusing on comorbid heart disease among patients with diabetes may be important. In addition, our results suggested clusters of countries with similar patterns of social and behavioral determinants of health. Patients in such countries may benefit from similar health promotion interventions. Our findings discourage policy makers and public health practitioners from implementing universal programs that assume social and behavioral determinants of well-being are the same across different settings. Our results may also explain why the same programs may have different effects on well-being of patients with diabetes across countries. Locally designed interventions may be superior to such rigid programs.

### Limitations

The current study had several limitations. Due to the cross sectional design, causative associations are not plausible from this study. In addition, cross–country differences in the validity of self-report of subjective health and chronic conditions cannot be ruled out. The study did not measure glucose control, type of diabetes, or mental health as other factors associated with subjective health of participants with diabetes. The study also ignores duration or complications of diabetes.

## Conclusion

Our study revealed major cross-country differences in social and behavioral determinants of well-being among patients with diabetes. Only comorbid heart disease was consistently associated with poor subjective health across all countries. The findings advocate for design and implementation of country–specific health promotion programs for patients with diabetes. Further research is needed on causes and consequences of cross-country variations in social and behavioral determinants of well-being among patients with chronic conditions.

## Competing interests

The author declares that he has no competing interests.
