# Correction: Cross-country variation in additive effects of socio-economics, health behaviors, and comorbidities on subjective health of patients with diabetes

**DOI:** 10.1186/2251-6581-13-75

**Published:** 2014-07-22

**Authors:** Shervin Assari

**Affiliations:** 1 Department of Health Behavior and Health Education, University of Michigan School of Public Health, 1415 Washington Heights, Ann Arbor, MI 48109-2029, USA; 2 Center for Research on Ethnicity, Culture and Health, School of Public Health, University of Michigan, 1415 Washington Heights, Ann Arbor, MI 48109-2029, USA

## Correction

The acknowledgment section of the manuscript [1] should read:

Publication of this manuscript was possible with the Cornely Fellowship awarded by the Center for Research on Ethnicity, Culture and Health (CRECH), University of Michigan School of Public Health to the author.
